# The influence of tobacco smoking on adhesion molecule profiles

**DOI:** 10.1186/1617-9625-1-1-7

**Published:** 2002-01-15

**Authors:** DA Scott, RM Palmer

**Affiliations:** 1 Department of Oral Biology, Faculty of Dentistry, University of Manitoba, 780 Bannatyne Ave, Winnipeg, MB, R3E 0W2, Canada; 2 Department of Periodontology and Preventive Dentistry, King's College London, UK

## Abstract

Sequential interactions between several adhesion molecules and their ligands regulate lymphocyte circulation and leukocyte recruitment to inflammatory foci. Adhesion molecules are, therefore, central and critical components of the immune and inflammatory system. We review the evidence that tobacco smoking dysregulates specific components of the adhesion cascade, which may be a common factor in several smoking-induced diseases. Smoking causes inappropriate leukocyte activation, leukocyte-endothelial adhesion, and neutrophil entrapment in the microvasculature, which may help initiate local tissue destruction. Appropriate inflammatory reactions may thus be compromised. In addition to smoke-induced alterations to membrane bound endothelial and leukocyte adhesion molecule expression, which may help explain the above phenomena, smoking has a profound influence on circulating adhesion molecule profiles, most notably sICAM-1 and specific sCD44 variants. Elevated concentrations of soluble adhesion molecules may simply reflect ongoing inflammatory processes. However, increasing evidence suggests that specific soluble adhesion molecules are immunomodulatory, and that alterations to soluble adhesion molecule profiles may represent a significant risk factor for several diverse diseases. This evidence is discussed herein.

## Introduction

Smoking leads to a generalised leukocytosis [1-7]; influences the production of most immunoglobulin classes and sub-classes [8-11]; can induce T cell anergy (failure to respond to antigen) [12-14]; effect vascular dynamics [15-19]; cause inappropriate priming and activation of monocytes and neutrophils [4,20,21]; abnormal platelet aggregation [22,23]; and a generalized increase in local and systemic inflammatory markers [21,24,25]. Therefore, there is considerable evidence that smoking exerts profound influences on multiple components of the immune and inflammatory system in humans, various aspects of which have been ably reviewed elsewhere [4,14,16,26,27]. This paper will focus on the influence of tobacco smoking on a group of molecules that function at the heart of the immune and inflammatory response – the cellular adhesion molecules.

## Adhesion Molecule Overview

**Figure 1:**
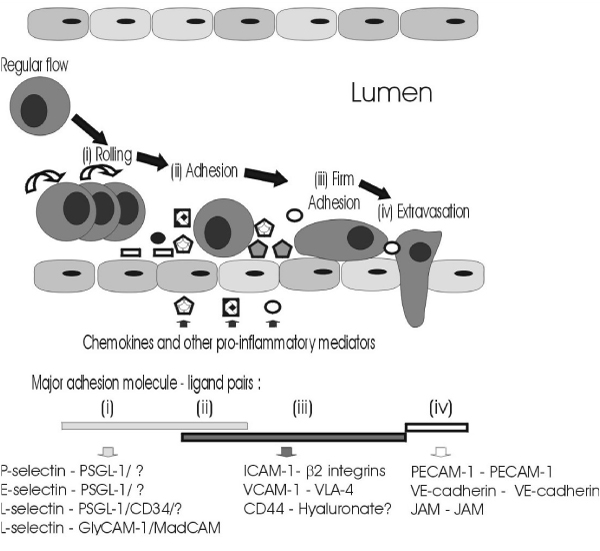
**Leukocyte adhesion and extravasation**. This figure has been adapted from references [28-31].

Adhesion molecules share the common physiological role of promoting cell-cell or cell-extracellular matrix adhesion, and many are multifunctional intracellular signalling molecules. The ability of lymphocytes (B cells and T cells) to circulate between the blood and secondary lymphoid tissue, and the movement of leukocytes (lymphocytes and other white blood cells, such as neutrophils and monocytes) from the systemic circulation to local sites of inflammation, are major and critical components of the immune response. Leukocytes must, therefore, interact with and cross endothelial barriers – either the high endothelial venules of the lymphatic system or the endothelial cells forming the microvasculature. The sequential processes of capture, rolling and adhesion of leukocytes to endothelial cells, and subsequent leukocyte extravasation and migration to specific sites, are mediated and controlled by a complex and overlapping series of interactions between adhesion molecules and their specific ligands – an adhesion cascade – as represented in Figure 1[28-31].

Essentially, leukocyte capture is mediated by P-selectin (CD62P) and L-selectin (CD62L). Rolling is mediated by P-selectin, L-selectin, and a third member of the selectin family of adhesion molecules, E-selectin (CD62E). Firm adhesion is mediated by interactions between intercellular adhesion molecule-1 (ICAM-1; CD54) and vascular cell adhesion molecule-1 (VCAM-1; CD106) and their ligands – the β_2 _integrins (particulary LFA-1 [α_L_β_2_; CD11a/CD18] and Mac-1 [α_M_β_2_; CD11b/CD18]) and very late-antigen-4 (VLA-4; CD49d), respectively – following further activation of the adhesion cascade by locally produced chemokines, and/or other stimuli, such as RANTES, fractaline, and stromal cell-derived factor-1α (SDF-1α) and SDF-1β, as reviewed in [31]. Extravasation through endothelial-endothelial cell junctions is less well understood. However, homophilic interactions of specific adhesion molecules [junctional adhesion molecules (JAM), platelet-endothelial cell adhesion molecule-1 (PECAM-1; CD31), and vascular endothelial cadherin (VE-cadherin; CDH5)] may play important roles in maintaining endothelial-endothelial cell adhesion, and these adhesion molecule interactions are probably disrupted in order to permit leukocyte transmigration [31-34]. Although the importance of another adhesion molecule, CD44, in lymphocyte homing has been known for some time, there is increasing evidence to suggest that CD44 is also involved in leukocyte adhesion and transmigration processes during inflammation [30,35]. Glycosylation, and other post-translational modifications; expression densities of adhesion molecules, and their ligands, on leukocytes and endothelial cells; activation-induced alterations in the affinity of adhesion molecules for specific ligands; intracellular signal transduction pathways initiated on rolling; and co-operation between various adhesion molecules, all influence leukocyte/endothelial interactions, as reviewed elsewhere [28,31,36-42]. Significant interference with leukocyte transmigration due to environmental, acquired, or genetic factor(s) may be expected to have profound consequences. A classic example is leukocyte adhesion deficiency type I, a rare genetic disorder defined by insufficient or non-functional CD18, the common chain of the β_2_-integrins, resulting in compromised leukocyte trafficking, reflected in severe and recurrent bacterial infections, and delayed wound healing. This paper will focus on the evidence that tobacco smoking also influences the expression and release of specific adhesion molecules.

### ICAM-1 and CD44

Of the major adhesion molecules, the evidence presented below will necessitate a particular focus on two – ICAM-1 and CD44. ICAM-1 is an integral membrane glycoprotein with five extracellular immunoglobulin-like domains and a short cytoplasmic tail, encoded by a 3.3 kb mRNA transcribed from a single gene with no alternate exons [43,44]. ICAM-1 is expressed constitutively by a wide variety of cell types, including endothelial cells. Basal expression of ICAM-1 on endothelial cells, and some leukocytes, can be augmented following induction by TNF-α, IL-1β, and IFN-γ, and other inflammatory mediators [38,45-49]. Recognised ligands of ICAM-1 include the β_2_-integrins of leukocytes, hyaluronate, sialophorin (CD43) and fibrinogen [50,51], reflecting the major role played by ICAM-1 immune and inflammatory regulation.

By way of introduction to a complex adhesion molecule family, human CD44 is a widely expressed family of glycoproteins, encoded by a single gene containing 20 exons. Numerous CD44 isoforms are generated through alternate splicing of pre-mRNA. However, all CD44 isoforms share the same N- and C-terminal sequences. The haematopoetic, or standard form, of CD44 (CD44H; CD44s) is a 248 amino acid protein that contains no variant exon-encoded peptide sequences. Thus CD44H is the smallest CD44 isoform, with a molecular mass of 80–95 kDa, more than half of which is due to post-translational glycosylation events. Splice variation and significant post-translational modifications result in a multifunctional group of CD44 adhesion molecules, with important functions in embryogenesis, lymphocyte activation and homing, angiogenesis, leukocyte extravasation, anti-apoptosis signalling, presentation of growth factors and proteases, and cell migration and proliferation, including during tumor metastasis [30,52-60]. Many of these activities appear to be mediated by interaction between the extracellular matrix component hyalouronate and CD44. However, several other CD44 ligands are recognised, including laminin, collagen, fibronectin, serglycin, osteopontin and aggrecan [57,61,62]. Many recent reviews address the structure, regulation, function, clinical significance, and therapeutic targeting of ICAM-1 [38,50,51,63-67] and CD44 [30,61-63,68-74].

## The Influence of Tobacco Smoking on Cell-Bound Adhesion Molecules

It has been known for some time that cigarette smoking can induce leukocyte-endothelial adhesion, microvascular and macrovascular entrapment of leukocytes, and leukocyte aggregation in humans and animal models [75-89]. Leukocyte-endothelial binding, and subsequent leukocyte-mediated tissue damage, is a central component of various smoking-associated inflammatory diseases, leading several groups to investigate the influence of tobacco use on adhesion molecule networks. This review will pay particular attention to **chronic obstructive pulmonary disease (COPD)**, direct cigarette smoke exposure being most intense in the lungs; **vascular diseases**, vascular endothelial cells and circulating leukocytes being chronically exposed to systemically distributed components and metabolites of smoke; and **chronic inflammatory periodontal disease (CIPD)**, with the periodontal tissues being both chronically exposed to systemic smoke components, and transiently exposed topically.

### Chronic Obstructive Pulmonary Disease

COPD is characterised by chronic coughing, obstruction of the peripheral airways, and destruction of lung surfaces (emphysema). Although not all smokers develop COPD, 90% of those who do develop COPD are smokers. As much of the pulmonary tissue destruction in COPD is thought to be due to the recruitment and activation of inflammatory cells, several researchers have examined the influence of smoking on adhesion molecule expression and release by the endothelial cells comprising the pulmonary vasculature, and recruited alveolar inflammatory cells. Tobacco smoking results in a significantly increased number of immature neutrophils in the systemic circulation, characteristic of chronic stimulation of bone marrow [7]. Accordingly, there is a small, but significant, increase in circulating neutrophil L-selectin expression in smokers [7]. Immature neutrophils are preferentially sequestered in the lung microvasculature [90], and pulmonary neutrophil entrapment is a recognised phenomenon in smokers [85]. Additionally, absolute neutrophil and macrophage numbers are increased in the alveolar space of smokers [24,88,91-93]. These observations point to a smoking-induced dysregulation of adhesion molecule networks in the pulmonary environment.

In support of this, Schaberg et al. [88] noted that a significantly higher proportion of alveolar macrophages from smokers expressed β_2_-integrin subunits than alveolar macrophages obtained by pulmonary lavage from non-smokers. The increase in β_2_-integrin expression correlated with increased numbers of macrophages in the pulmonary environment. Increased binding of alveolar macrophages from smokers to TNF-α stimulated human umbilical vein endothelial cells (HUVECs) was mitigated by either pre-treatment of the endothelial cells with antibodies to ICAM-1, or pre-treating alveolar macrophages with antibodies to CD18. Therefore, smoking may contribute to increased ICAM-1/β_2_-integrin-dependent recruitment of inflammatory cells to the lungs. Lensmar et al. [94] reported that while the sputum of smokers (n = 9) contained more macrophages than the sputum of non-smokers (n = 7), the percentage of macrophages expressing ICAM-1 was significantly lower in smokers. However, upregulation of endothelial ICAM-1 expression is likely to be a more important limiting factor in terms of leukocyte recruitment.

In studies of the pulmonary endothelium, there is some evidence that ICAM-1 expression by bronchial vessels may not be affected by smoking [95]. However, Schaberg et al. [93] went on to observe a large increase in ICAM-1, but not P-selectin, E-selectin, or VCAM-1, expression by the endothelium of peripheral pulmonary vessels in smokers, compared to non-smokers. Schaberg et al. [93] also noted a strong correlation between cumulative tobacco smoke exposure (pack-years) and the percentage of ICAM-1-positive pulmonary vessels, consistent with a role for smoking in increased pulmonary recruitment of leukocytes through the dysregulation of adhesion molecule networks.

As only some smokers develop COPD, there is an obvious interest in defining the characteristics of smokers at enhanced risk of developing the disease [96]. To this end, Maestrelli et al. [97] reported that the numbers of neutrophils expressing CD11b and CD18, but not CD11a or CD11c, were increased in smoking subjects (n = 33) with airway obstruction, compared to smokers without airway obstruction, and hypothesised that CD11b/CD18 expression by sputum neutrophils may represent a marker for the development of chronic airway obstruction among smokers. Noguera et al. [98] investigated the characteristics of circulating neutrophils in subjects with COPD and in smokers and non-smokers without COPD. None of the COPD subjects were current smokers, although they had a total cumulative tobacco exposure similar to the smoking group (50 pack-years). There were no significant differences in Mac-1, LFA-1, or L-selectin expression by TNF-α stimulated or unstimulated neutrophils isolated from smokers or non-smokers with normal forced-expiratory volume. However, expression of Mac-1 on neutrophils was increased in those with COPD, compared to either healthy group (smokers or non-smokers), augmented by an increased respiratory burst, leading the authors to suggest that neutrophil dysfunction in COPD subjects may not be directly caused by smoking, but, rather, may represent a characteristic of COPD. It is also possible that smoking may not affect all smokers equally, and that a differential effect on CD18/CD11b expression could discriminate smokers at increased risk of developing COPD. However, a recent comparison of adhesion molecule expression profiles on leukocyte (L-selectin, VLA-4, and the three β_2_-integrin heterodimers), endothelial and epithelial (E-selectin, P-selectin, VCAM-1, ICAM-1, and ICAM-2) cell surfaces in freshly resected lungs from smokers with airways obstruction (n = 10) and smokers with normal lung function (n = 10) revealed no significant differences in adhesion molecule profiles between diseased and healthy groups [99]. The authors, therefore, concluded that development of airways obstruction in smokers could not be explained by differences in the expression of adhesion molecules known to be involved in the control of cell traffic in the lung.

Other investigations have monitored the effect of smoking on bronchial epithelial cells. Small-airway epithelial cells harvested from smokers exhibited significantly elevated ICAM-1 mRNA levels, compared to non-smokers [100]. Correspondingly, a dramatic increase in ICAM-1 release from the surface of cultured small-airway epithelial cells of smokers compared to cells from non-smokers (356 pg 10^6 ^cells^-1 ^vs 113 pg 10^6^cells^-1^) was noted. No such differences were noted in epithelial cells isolated from the main bronchi of smokers and non-smokers. Similarly, di Stefano et al. [95] found no difference in ICAM-1 expression on the bronchial epithelium of smokers and non-smokers. Cigarette smoke exposure resulted in a significant increase (up to 175%) in sICAM-1 release from primary explant cultures of human bronchial epithelial cells obtained from never-smokers, or smokers with COPD, but not in cultured bronchial epithelial cells from smokers with normal pulmonary function [101]. As similar results were noted for concentrations of the proinflammatory cytokine IL-1β, this suggests that not all smokers are equally susceptible to cigarette-smoke induced alterations to the pulmonary inflammatory response.

### Vascular diseases

The pathogenesis of atherosclerosis, the precursor to most acute coronary syndromes and strokes, includes the activation of vascular endothelial cells, and the recruitment of inflammatory cells, predominantly macrophages, to the vessel wall [35,102,103]. Leukocyte-endothelial recruitment is adhesion molecule-dependent, and smoking is a major risk factor for vascular diseases. Therefore, attention has inevitably turned to examinations of the influence of tobacco smoking on adhesion molecule expression by vascular endothelial cells. Indeed, tobacco smoking has long been known to induce leukocyte-endothelial adhesion and leukocyte entrapment, as noted earlier.

Monocyte-endothelial interaction is clearly multifactorial. However, endothelial ICAM-1 expression is likely to exert a strong influence on such cell-cell adhesions. Adams et al. [102] were able to show upregulation of ICAM-1 (but not VCAM-1 or E-selectin) expression on HUVECs exposed to smokers' serum, and to confirm increased monocyte-endothelium adhesion in smokers selected to exhibit no other major risk factor for vascular disease. Nicotine has been shown to induce an increase in VCAM-1 mRNA synthesis, but not E-selectin, in primary human coronary artery endothelial cells, but a 1.6 fold elevation required 24 hr exposure to a concentration of nicotine as high as 10^-5^M (or 1.6 μg ml^-1^), which is higher than normal physiological exposure levels [17,104,105]. Weber et al. [89] determined that increased monocyte-endothelial adhesion in smokers is CD11b-dependent, through the use of a blocking anti-CD11b antibody. However, CD11b was not increased on the surface of freshly isolated monocytes. This indicates that smoking may lead to increased monocyte-HUVEC adhesion by influencing an endothelial Mac-1 ligand, with ICAM-1 the prime candidate, or that smoking may induce conformational, or other, alterations to Mac-1, leading to increased affinity for ICAM-1. Indeed, increased expression of CD11b may not be required or sufficient for increased leukocyte-endothelial adhesion [89,106,107].

Neutrophils contain large granular stores of CD11b that can be rapidly translocated to the surface on activation [108,109]. Therefore, tobacco-induced translocation of CD11b could partly explain enhanced neutrophil adhesion in smokers. Recently, Koether et al. [20] exposed whole blood or neutrophils isolated from non-smokers to cigarette smoke condensate (CSC), and observed a rapid (minutes) 2.5 to 3 fold increase in CD18/CD11b expression on the neutrophil surface. Other studies have also shown an increased tobacco-induced expression of cell surface β_2_-integrins by leukocytes in *in vitro *studies [33,80,110]. Thus, up-regulation of CD11b/CD18 in primed neutrophils, coupled with endothelial ICAM-1 expression, would be expected to contribute to inappropriate neutrophil adhesion in smokers in cardiovascular, pulmonary, or other environments, with potential ramifications to local tissue integrity. However, this specific issue is not clearcut. Most *in vivo*, and some *in vitro*, studies in humans have not shown a difference between expression levels of β_2_-integrins on neutrophils and monocytes and of smokers and non-smokers [4,7,105,111], which hints at a need for consideration of cell conditioning to tobacco smoke, and/or physiologically relevant dosing in *in vitro *models. Typical serum nicotine levels in smokers are in the region of 30 ng ml^-1^[17,104,105].

Shen et al. started the important task of dissecting the signalling pathways that underlie tobacco-induced monocyte-endothelial adhesion [33]. Cigarette smoke condensate (CSC) was shown to induce surface expression of ICAM-1, VCAM-1, and E-selectin on HUVECs and bovine aorta endothelial cells. Nicotine alone, even at 25 mg ml-1, did not influence endothelial adhesion molecule profiles. Inhibitors of protein synthesis (cycloheximide), transcription (actinomycin D) and protein kinase C (GF 109203X and chelerythrine), established that increased endothelial adhesion molecule expression required de novo adhesion molecule production, via a PKC-dependent pathway. NF-κB, a transcription factor, binds to a consensus site in specific adhesion molecule genes. Shen et al. [33] established the importance of CSC-induced NF-κB activation in the upregulation of endothelial adhesion molecule expression. Furthermore, CSC increased the migration of monocytic cells across HUVECs and bovine aorta endothelial cells, concomitant with an up to 10-fold increase in PECAM-1 phosphorylation, which was inhibited by PKC inhibitors. Unfortunately, such studies on the molecular mechanisms by which smoking may dysregulate the expression and release of specific adhesion molecules are rare, and there is an obvious need for a concerted research effort in this area.

Several groups have attempted to reduce smoking-induced leukocyte-endothelial adhesion in smokers by pharmacological intervention with anti-inflammatory agents. Smoking-induced monocyte-endothelial adhesion in humans was acutely abrogated to a significant degree by a single 7 g oral administration of L-arginine, but not vitamin C, suggesting a role for nitric oxide [102]. Weber et al. [89] showed that ten days of Vitamin C supplementation was successful in reducing monocyte-endothelial adhesion in smokers. Zapolska-Downar et al. [21] noted that oral administration of the non-steroidal anti-inflammatory drug ibuprofen reduced monocyte-HUVEC adhesion in smokers.

Adhesion molecule-mediated platelet aggregation represents a mechanism contributing to acute coronary events in smokers [22]. Accordingly, it has been shown that P-selectin expression is generally increased on the surface of platelets in smokers, a process that requires translocation of P-selectin from the intracellular α-granules [22,112]. In vivo platelet activation, and the associated alterations in cell surface presentation of P-selectin molecules, can occur immediately after smoking a cigarette [22]. The increased surface of P-selectin on platelets is not affected by aspirin administration (2 weeks at 100 mg/day) [112].

### Chronic inflammatory periodontal disease

Chronic inflammatory periodontal disease (CIPD) is a common disease of the supporting tissues of the teeth, resulting from a complex interaction between plaque bacteria and the host response. Like vascular disease and COPD, tobacco smoking is also a major risk factor for the development, progression, and exacerbation of periodontitis [113-116].

ICAM-1 expression is increased in the gingival vasculature during episodes of experimental gingivitis, -induced on cessation of normal oral hygiene procedures-coincident with peak measurements of IL-1 in the gingival crevicular fluid (GCF; a serum-derived fluid that exudes from the gingival sulcus) [117]. Rezavandi et al. [18] examined adhesion molecule profiles in periodontal tissues taken from smokers and non-smokers undergoing surgical treatment for CIPD. Compared to non-inflamed areas, the proportion of the microvasculature expressing ICAM-1 and E-selectin was significantly increased in inflamed areas of tissues, irrespective of smoking status. However, although smokers had thus retained the ability to upregulate adhesion molecule expression, an integral part of the inflammatory response, other signs of smoking-induced immune dysregulation in the gingival microvasculature were apparent. There was no statistically significant difference in the number of small blood vessels, detected by immunostaining of von Willebrand factor, in inflamed and non-inflamed areas of the gingival tissues of smokers. In other words, the normal angiogenic response that helps to define inflammation was compromised.

**Figure 2:**
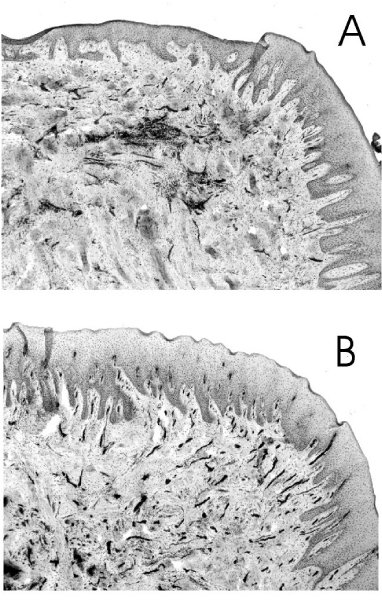
**Expression of ICAM-1 in the gingival tissues of a smoker and non-smoker with chronic inflammatory periodontal disease**. Typical histological sections of gingival tissue from a smoker (A) and a non-smoker (B) with CIPD. Tissue sections were labelled with monoclonal antibodies to ICAM-1, visualised with streptavidin-biotin peroxidase complex developed with diaminobenzidine/hydrogen peroxide, and counter-stained with haematoxylin. ICAM-1 positive cells (dark staining) are predominantly endothelial. The percentage of ICAM-1-positive vessels of the gingival microvasculature was determined following immunostaining of von Willebrand factor, an endothelial marker, in adjacent sections.

Rezavandi et al. [18] also noted that in histologically normal, non-inflamed periodontal tissues, the percentage of vessels expressing ICAM-1 was reduced in smokers. Thus, basal endothelial ICAM-1 expression levels may be compromised. Typical expression profiles of ICAM-1 in the gingival tissues of a smoker and non-smoker with CIPD are presented in Figure 2. As mentioned earlier, constituents of tobacco smoke can induce ICAM-1 expression on endothelial cells [33,80,93,102]. One possible outcome of long-term tobacco smoke exposure is tolerance, with respect to ICAM-1 expression. It is also a possibility that the reduced level of ICAM-1 expression in normal gingival tissues could reflect ongoing shedding of membrane-bound ICAM-1. Smokers are, indeed, known to carry a high load of a soluble form(s) of ICAM-1 in the systemic circulation [105,118], a topic we shall re-address shortly.

In contrast to the pulmonary alveolar space, the absolute number of neutrophils in the GCF or oral cavity fluids of smokers is comparable or lower in smokers, compared to non-smokers [119,120]. This is despite the increased number of neutrophils in the systemic circulation of smokers [5,7]. The reduced angiogenic response and compromised endothelial ICAM-1 expression profiles in the gingival tissues of smokers may help explain this initially surprising observation.

In summary, there is growing evidence that smoking influences tissue and cellular adhesion molecule expression profiles in several smoking-induced diseases: COPD; vascular diseases; and CIPD. However, such studies are at a preliminary stage, all tissues do not appear to respond equally, and the *in vivo *evidence for smoking-induced cell-associated adhesion molecule dysregulation has been obtained primarily with small numbers of patients. Further research into the influence of tobacco on adhesion molecules in smoking-induced inflammatory disease is warranted and necessary.

## Soluble Adhesion Molecules

**Figure 3:**
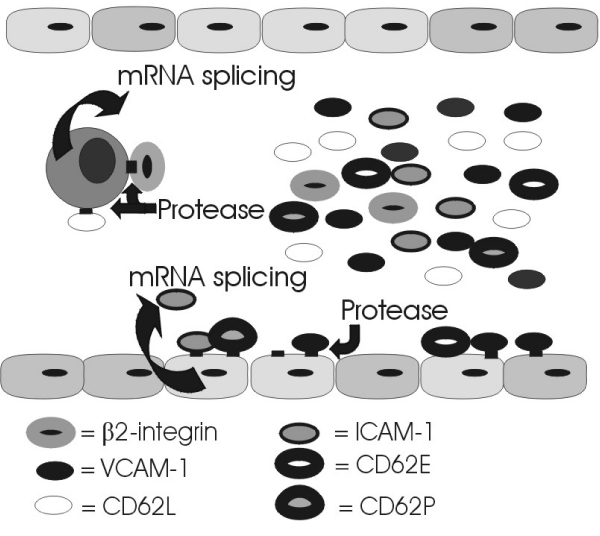
**Release of soluble adhesion molecules**.

Soluble forms of many adhesion molecules are recognised (see Figure 3), but the relevance of these circulating forms of adhesion molecules is not clearly understood. Specific circulating adhesion molecules are proteolytically released from the cell surface of activated immune cells [45,48,121], therefore, these circulating adhesion molecules may simply reflect general immune function. However, there is convincing evidence that certain soluble adhesion molecules remain bioactive and have the potential to interfere with a variety of immunologic/inflammatory processes [29,122]. As noted early on by Gearing and Newman [29], there are two obvious means by which soluble forms of adhesion molecules may have significant physiological influence – (i) by competitive inhibition of cell-cell interactions and, (ii) by binding to the appropriate ligand on the surface of a cell, thereby eliciting a response.

### sICAM-1

The evidence that tobacco smoking leads to a significant increase in circulating levels of sICAM-1 is overwhelming. In an investigation into the relationship between local (periodontal) and systemic inflammation, a significant elevation of sICAM-1 in smoking subjects, independent of periodontal status, was noted [118]. This observation has since been confirmed by many authors [105,123-135]. In the pulmonary environment, involuntary cigarette smoke exposure ("environmental" or "passive" smoking) has been associated with significantly increased ICAM-1 concentrations in the bronchoalveolar lavage fluid of children [136]. Rumalla et al. [137] noticed a 10 fold elevation in sICAM-1 levels in the pulmonary lavage fluids of some individual smokers.

Cigarette smoking does not seem to lead to an acute increase in circulating sICAM-1 levels [105,125]. Thus, raised circulating sICAM-1 levels in smokers could reflect tobacco-induced vascular damage, or other underlying pathologies. However, further studies have since established that the influence of smoking on sICAM-1 levels is dose-dependent and reversible. There is a strong correlation between sICAM-1 levels and several quantitative indices of recent tobacco smoke intake – expired-air CO levels [138]; plasma and serum cotinine concentrations [105,138]; a composite index of tobacco intake [138]; and the systemic nicotine intake from a single cigarette [105]. Systemic sICAM-1 levels decline rapidly on smoking cessation to levels approaching that of non-smokers [125,138]. Much of this recovery occurs in the first four weeks following biochemically-validated cessation (unpublished data). Taken as a whole these data provide strong evidence that tobacco smoking is a direct causative agent of a systemically increased sICAM-1 burden. The decline in sICAM-1 levels noted on cessation is not compromised by high-dosage nicotine replacement therapy, in the form of transdermal nicotine patches (unpublished data). Therefore, it is a component (or metabolite) of tobacco smoke other than nicotine (or cotinine) that is responsible for elevated sICAM-1 concentrations in smokers. Typical concentrations of circulating adhesion molecules are presented in Table 1.

**Table 1:** Typical systemic adhesion molecule concentrations (ng ml^-1^) in age and gender matched smokers, non-smokers, and ex-smokers

Adhesion molecule	Non-smokers	Smokers	Ex-smokers**
sP-selectin	70 – 150	60 – 145	-
sL-selectin	760 – 1200	940 – 1100	-
sE-selectin	40 – 45	40 – 45	-
sICAM-1	220 – 300*	320 – 380**	240 (-71)*
sVCAM-1	500 – 610	500 – 595	-
sPECAM-1	25	30	-
sCD44v5	35*	60**	40 (-13)*
sCD44v6	140*	265**	170 (-62)*

* p < 0.05.

* * Ex-smokers taken from the pan-European CEASE trial [139]. Ex-smokers smoked ≥ 15 cigarettes per day at baseline (plasma cotinine ≥ 50 ng ml^-1^); and ceased tobacco use for 52 weeks. Tobacco cessation was validated biochemically (expired-air CO < 10 ppm and plasma cotinine <15 ng ml^-1^) at regular intervals. Ex-smokers were matched for baseline tobacco consumption, gender and age with continuing smokers. Drop in adhesion molecule concentration (ng ml^-1^) over one year given in brackets.

Data taken from [105,118,123,124,127,134,138-141].

### sICAM-1 and disease

A growing number of studies report a significant association between elevated sICAM-1 levels, and the presence of most vascular diseases; the prediction of future adverse events; and poor prognosis [128,131,134,142-149]. Thus, elevated sICAM-1 levels may have serious consequences. Serum sICAM-1 concentrations are also reported to be significantly elevated in subjects with other diverse disease entities, including non-small-cell lung cancer and other malignancies [127,150], diabetes [29,151], cystic fibrosis [152], inflammatory bowel diseases [153], bronchial asthma [154], allergic alveolitis [155,156], and several other inflammatory diseases. However, a majority of such studies have not considered smoking habits. Therefore some degree of re-evaluation of sICAM-1 as a disease marker may be required in light of the profound, and reversible, influence of tobacco smoke on sICAM-1.

Blann et al. [157] reported that increased sICAM-1 concentration was only a weak predictor of disease progression in peripheral atherosclerosis, when the study population was balanced for age, gender, and smoking status. Wallen et al. [134] observed that while elevated sICAM-1 levels in those with angina pectoris was associated with cardiovascular death, or non-fatal myocardial infarction (354 ng ml^-1^, n = 7) compared to those who remained event free (282 ng ml^-1^, n = 86), clinical risk factors, including smoking (57% vs 21%), were more prevalent in those with a poor outcome. Additionally, mean sICAM-1 was significantly raised in smokers, compared to never-smokers, to a comparable extent to that noted between coronary event and event free groups. Similarly, O'Malley et al. [130] observed a significant increase in sICAM-1 levels in a group of individuals with ischaemic heart disease, both at time of presentation with chest pain, and three months later, compared to healthy controls. Therefore, sICAM-1 levels where not influenced by the acute event. However, for the purposes of this review it is most interesting that sICAM-1 elevation was confounded by cigarette smoking. Fassbender et al. [126] monitored soluble adhesion molecule profiles in 173 subjects with cerebrovascular disaeases, and 67 controls. Although, there was an increase in sICAM-1 levels in the total population with cerebrovascular disease compared to controls (275 ng ml^-1 ^vs 260 ng ml^-1^, respectively), the diseased population contained twice the number of smokers, and the authors note that sICAM-1 levels were significantly increased in smoking subjects. Again, Rifai et al. [158] examined sICAM-1 levels in the plasma of 100 men with angiographically documented coronary heart disease, and 100 healthy controls matched for age and smoking status. Under this experimental strategy, there was no significant difference in median sICAM-1 concentrations between the diseased (335 ng ml^-1^) and healthy groups (339 ng ml^-1^).

Some studies certainly suggest that elevated sICAM-1 is a suitable marker of vascular disease status and a risk factor for future acute vascular events [131,146,149], and that these significant associations are not confounded by smoking habits. Nevertheless, it has been clearly established that a dramatic, dose-dependent increase in circulating sICAM-1 levels is one consequence of tobacco smoking. Therefore smokers will be more likely to be in the upper quartile of sICAM-1 levels, where increased risk of vascular disease is most evident [146]. Indeed, the influence of smoking on sICAM-1 concentration is commonly reported to be as great, or greater [118,126,158] as that ascribed to disease [126,130,158].

**Figure 4:**
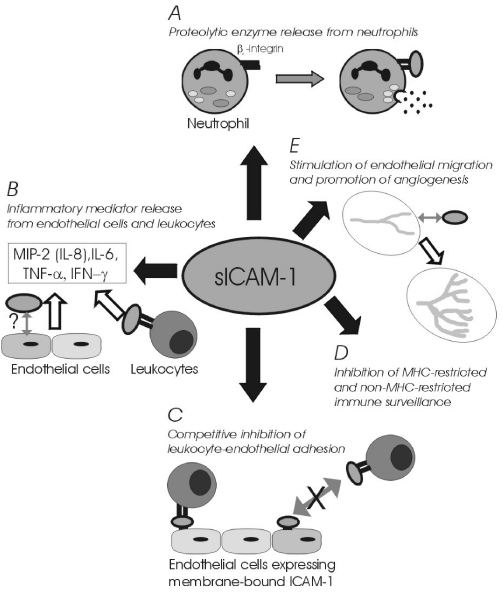
**Immunomodulatory functions of sICAM-1**. The following references were considered in the construction of this illustration. A. [159-161]. B. [162-165]. C. [47,107,166-170]. D. [171-176]. E. [177].

Recently, Becker et al. [149] have suggested that the significant relationship between elevated systemic sICAM-1 and vascular disease is independent of renal function and major risk factors such as clinical or sub-clinical atherosclerosis (prior vascular disease or ankle-brachial pressure index), endothelial activation (von Willebrand factor), inflammation (C-reactive protein), or elevated sVCAM-1. Thus a causative link between sICAM-1 and vascular disease has yet to be firmly established. While it is likely that underlying inflammatory disease(s) will contribute to some extent to the systemic sICAM-1 load, the possibility that the tobacco-induced sICAM-1 burden may directly influence the immune response, and thus represent a common factor in tobacco-induced diseases, is deserving of attention. To this end, the immunomodulatory effects attributed to sICAM-1 that may be relevant to various disease processes are summarised in Figure 4. Briefly, stimulation of proteloytic enzyme release from granulocytes, which has been proposed as a mediator of multiple organ failure [159], could make a major contribution to tissue breakdown; stimulation of inflammatory mediator release may be expected to potentiate the inflammatory response; competitive inhibition of leukocyte-endothelial interactions may be expected to compromise migration of activated leukocytes; inhibition of immune surveillance could abet tumor cells in evading detection; and promotion of angiogenesis may aid tumor growth, and have obvious relevance to other angiogenesis-dependent diseases. Detailed explanations of these proposed bioactive sICAM-1 properties can be obtained from the references provided in the figure legend [47,159-177].

The antioxidant capacity of smokers is compromised [178]. The influence of antioxidant therapy on smoking-induced endothelial-leukocyte interactions was discussed briefly earlier. We are currently in the final stages of a study examining the short-term influence of high-dose vitamin C on circulating ICAM-1 levels. However, since embarking on this study an independent research group [133] has reported that the elevation of serum sICAM-1 in smokers is not abrogated by long-term treatment with a different class of antioxidant molecule-α-tocopherol (2 years at 400 IU dL/day). sICAM-1 levels were reduced in healthy men by glucocorticoid (dexamethasone) treatment [179].

### Molecular characteristics of sICAM-1

sICAM-1 exists as a 80–105 kDa monomeric protein [50,51,180,181], and as an unconfirmed immunoreactively distinct, and essentially uncharacterised, 130 kDa protein [182]. Evidence for alternate splicing resulting in two mRNA species directly encoding either sICAM-1 or membrane-bound ICAM-1 in humans has been presented [183]. However, the majority of studies have found no evidence for alternate mRNA species in humans, and the predominant theory is that sICAM-1 is released from the cell surface following proteolysis of the cell-bound form, at a defined site [45,48,49,184]. Dimeric ICAM-1 exhibits significantly higher affinity for its major ligand (LFA-1) than monomeric ICAM-1 [185,186]. Young et al. [187] purified sICAM-1 from normal human serum and the urine of patients with bladder cancer by immunoaffinity chromatography and detected only monomeric sICAM-1, which could not block LFA-1/ICAM-1-dependent cell-mediated cytolosis of bladder tumour target cells. However, others have described sICAM-1 dimers and multimers *in vivo *[180,181]. Transcriptional regulation of ICAM-1 is particularly complex, with unusual post-transcriptional control mechanisms described [50,51,66]. Cell-specific regulatory mechanisms and cell-specific post-translational modifications of ICAM-1 proteins may affect ICAM-1 function [184,188,189]. However, to the best of our knowledge, the character, and source, of ICAM-1 molecules elevated in smokers has not yet been investigated. Clearly, clarification of the specific molecular characteristics of sICAM-1 in smokers would represent a huge step towards an understanding the potential role of sICAM-1 in smoking-induced diseases. Equally, there is a pressing need to understand the mechanisms by which components, or metabolites, of tobacco smoke induce the upregulation and shedding of sICAM-1 molecules from the endothelium, and other cellular sources. Interestingly, despite elevated systemic sICAM-1 concentration noted in smokers, there is a consistent decrease, approaching four fold, in GCF ICAM-1 levels in smokers with CIPD [190]. No difference between serum and GCF sICAM-1 concentration is apparent in non-smokers. Thus, ICAM-1 molecules are inhibited in their passage from the periodontal microvasculature or through the periodontal tissues. This indirectly implies the sICAM-1 molecules in smokers are active, and may be interacting with to be determined ligands. However, a reduced angiogenic response in smokers is another possible explanation for these observations.

### sCD44

In an investigation designed to examine the potential of specific reputed tumour-associated circulating sCD44 isoforms to act as biomarkers in certain malignancies, Kittl et al. [140] observed a significant elevation in the mean concentration of sCD44 containing the product of exon 5 (sCD44v5) and sCD44 containing the product of exon 6 (sCD44v6), in self-reported smokers, compared to self-reported non-smokers. Total sCD44 has since been shown to be elevated in the blood of smokers, compared to non-smokers [191], to a small, but significant, extent. Like sICAM-1, the elevations in CD44v5 and v6, but not total CD44, are dose-dependent and reversible [141]. Smoking cessation led to a reduction in concentrations of both sCD44v5 and sCD44v6 (-13 ng ml^-1 ^and -62 ng ml^-1^, repectively) to levels approaching those reported in non-smokers [140,141]. Additionally, sCD44 recovery was not influenced by transdermal nicotine replacement therapy (unpublished results).

Alterations to circulating sCD44 profiles patterns have been extensively attributed diagnostic and prognostic potential in several malignancies, including, but not limited to, non-Hodgkin's lymphoma, breast, gastric, and colon cancer [61,72,192-199]. Soluble CD44 is also known to be raised in subjects with particular inflammatory conditions [200-202]. However, the majority of studies that have examined the potential utility of sCD44 isoforms as disease biomarkers have not considered smoking status. In light of the recent evidence that smoking exerts a profound dose-dependent and reversible influence on systemic concentrations of specific sCD44 variant molecules, then it may be necessary to re-evaluate ascribed diagnostic and prognostic specificities in certain inflammatory and malignant diseases. Cellular and tissue CD44 isoform expression patterns have also been widely proposed as markers of tumor growth, metastatic potential, and poor prognosis in several malignancies, including lung cancer [53,60,203-210]. Alternations to CD44 profiles have also been noted in non-cancerous, but pre-neoplastic, lung tissues [210]. There is, however, evidence that tobacco can induce alterations to cell-bound CD44 profiles in normal cells. Nicotine and its primary metabolite – cotinine – have both been shown to alter CD44 expression profiles on lung microvascular endothelial cells (LEISVO), and bone marrow-derived (STR-12) endothelial cells [211]. Therefore, it may be useful to address the influence of tobacco on the expression of membrane-bound CD44 isoforms *in situ *in a definitive manner in future studies.

Soluble CD44 was first reported in serum over twenty years ago [212]. Despite this, the physiological relevance of sCD44 molecules is unclear. However, by analogy with sICAM-1, it is entirely possible that circulating CD44 molecules could be immunomodulatory. There is some evidence to support this theory. In an *in vitro *system, sCD44 (in liposomes) was able to partially suppress T cell activation [213]. Recently, a novel sCD44 splice variant (CD44RC) was cloned that dramatically enhanced the hyaluronan binding activity of cell surface CD44 [214]. This unusual and unexpected observation has been suggested to result from CD44RC binding to chondroitin sulfate side-chains attached to cell surface CD44. Therefore, a multivalent complex with increased avidity for hyaluronan is generated. In addition to this functional originality, CD44RC is produced in a, so far, unique manner – splicing of the 3' end of CD44 exon 2 into an internal site within exon 18, resulting in an altered reading frame and, thus, a novel CD44 isoform. Other studies have suggested that sCD44 variants can bind hyaluronate, and that the affinity of binding is determined by specific glycosylations [196,215]. Specific sCD44 molecules can bind fibronectin [196]. sCD44, in serum, has also been shown to inhibit human peripheral blood lymphocyte binding to endothelial cells in frozen tonsil sections [196]. Thus, specific sCD44 molecules are bioactive and, potentially, immunomodulatory.

Metastasis to the lung following intravenous delivery of TA3/St murine mammary carcinoma cells is prevented when the carcinoma cells are transfected with sCD44-encoding cDNA, where sCD44 probably acts as a competitive inhibitor of cell surface CD44-hyaluronate interactions [216]. Indeed, sCD44 transfection was subsequently shown to prevent hyaluronate-mediated clustering of CD44 on tumour cell surface [60]. Peterson et al. [217] inhibited murine mammary carcinoma cell growth through transfection with cDNA encoding hyaluronate-binding sCD44v8-v10 or sCD44v6-v10. Other studies have also indicated that sCD44 may interfere with the growth and metastasis of specific tumour cells in mice [217-220]. Suppression of tumour formation by a human sCD44 fusion protein has again been demonstrated lately [221], and a soluble CD44-immunoglobulin fusion protein was able to block the migration of a CD44H-transfected human melanoma cell line across a hyaluronate-coated surface [222]. Interestingly, CD44 may act as a tumor cell surface anchor for MMP-9, a matrix metalloproteinase, which may contribute to collagen degradation and contribute to tumor invasiveness [60]. sCD44 can disrupt CD44/MMP-9 clusters and inhibit tumor metastasis *in vivo *[60].

Alternate splicing of the CD44 pre-mRNA permits the subsequent translation of a plethora of potential CD44 variant proteins, whose function may be further modified by extensive and varied post-translational modifications. The molecular characteristics of smoking-influenced CD44 proteins have yet to be ascertained. CD44 has proposed roles in leukocyte-endothelial interactions, there is increasing evidence of several potential mechanisms by which CD44 may play an aetiological role in certain cancers and vascular diseases [35,53,55,59,60], there are reputed diagnostic associations between CD44 profiles and various diseases, and there are undoubted connections between tobacco use and specific cancers and certain life-threatening inflammatory processes. Consideration of the available data shows that there is a need to further develop our understanding of the influence of tobacco smoking on the CD44 gene, and encoded variant CD44 protein family, both as a means of properly attributing any diagnostic and prognostic significance to CD44 molecules, and perhaps, in order to unravel any mechanisms of tobacco-induced disease that may be, partially at least, CD44-mediated.

### Other soluble adhesion molecules

Blann et al. [157] reported that, compared to appropriate control groups, sVCAM-1 is significantly elevated in the serum of smokers with peripheral artery disease, but not healthy smokers, implying an indirect relationship. Elsewhere, much of the available evidence suggests that tobacco smoking does not influence sVCAM-1 concentrations [134,142,223]. Osterud et al. [224] found that sVCAM-1 levels were actually lower in male smokers, compared to non-smokers, but this was not true for females, or combined genders. Equally, and on balance, smoking does not appear to influence systemic concentrations of the selectins [105,112,118,151,225-227], or PECAM-1 [105,224,228]. However, there is some evidence to suggest that smoking could influence sP-selectin levels. Blann et al. [229] observed a significant rise sP-selectin levels in smokers. However, the smokers and non-smoking subjects were not matched for age or disease status (deep venous thrombosis), and the difference was not impressive. This is broadly in agreement with previous reports by the same authors, who have acknowledged that any potential relationship between smoking and sP-selectin is weak [124,230-232]. Osterud et al. [224] found a small, but significant, rise in sP-selectin in the serum of women smokers, compared to non-smokers, that was not apparent in the male or in the total study population. Limited evidence suggests that smoking could influence circulating sE-selectin concentrations. Kitamura et al. [233] observed a significant increase in serum sE-selectin levels in heavy smokers (20–40 cigarettes day^-1^; 92 ng ml^-1^), compared to non-smokers (67 ng ml^-1^). However, this observation was made in subjects with pustulosis palmaris et plantaris, a dermatological disease that seems to exert a strong influence on sE-selectin in serum, and it is not clear how the incidence of this confounding condition was distributed between the smoking and non-smoking groups. Thus, the balance of evidence suggests that if circulating profiles of other major adhesion molecules are indeed influenced by tobacco smoking, then this influence is certainly not as profound as the dramatic effects seen in sICAM-1 and some sCD44 variants.

## Concluding Remarks

Research into the relationship between tobacco use and adhesion molecule networks is at an early stage. Smoking directly causes an increase in soluble ICAM-1 and specific CD44 variant concentrations in the systemic circulation, diluting several previous diagnostic and prognostic attributions. Both sICAM-1 and CD44 are considered to possess several immunomodulatory properties. Clarification of the specific sources and molecular characteristics of smoking-influenced sICAM-1 and sCD44 protein should provide insight into potential functions of soluble adhesion molecules in tobacco-induced diseases. The influence of tobacco on the cellular and tissue distribution of cell-bound adhesion molecules is less clear. Smoking status should certainly be considered in studies that examine potential prognostic and diagnostic assignations of significance to circulating adhesion molecule profiles and membrane-bound expression in cells and tissues. This does not occur in the majority of studies, even in diseases were the aetiological significance of smoking is unequivocal, such as neoplastic and malignant lung tissues and COPD. Although there is extensive, and growing, knowledge of the molecular mechanisms that regulate transcription, expression, and shedding of ICAM-1, CD44, and other adhesion molecules, studies that address how tobacco smoke may influence these control networks are minimal. Adhesion molecules function at the heart of the immune and inflammatory response. Dysregulation of these critical molecules may represent a common mechanism(s) underlying susceptibility to a variety smoking-induced diseases.

## Abbreviations used

CIPD: chronic inflammatory periodontal disease; COPD: chronic obstructive pulmonary disease; GCF: gingival crevicular fluid; HUVEC: human umbilical vein endothelial cells; ICAM-1: intercellular adhesion molecule-1 (CD54); LFA-1: lymphocyte functional antigen-1 (CD11a/CD18; (α_L_β_2_); Mac-1: CD11b/CD18 (α_M_β_2_); PECAM-1: platelet-endothelial cell adhesion molecule-1; VCAM-1: vascular cell adhesion molecule-1 (CD106).

## Competing interests

The authors declare that they have no competing interests.
